# Nicotine attenuates the effect of HIV-1 proteins on the neural circuits of working and contextual memories

**DOI:** 10.1186/s13041-015-0134-x

**Published:** 2015-07-24

**Authors:** Tanseli Nesil, Junran Cao, Zhongli Yang, Sulie L. Chang, Ming D. Li

**Affiliations:** Department of Psychiatry and Neurobehavioral Sciences, University of Virginia, 450 Ray C Hunt Drive, Suite G-170, Charlottesville, VA 22903 USA; Institute of NeuroImmune Pharmacology, Seton Hall University, South Orange, NJ USA; Department of Biological Sciences, Seton Hall University, South Orange, NJ USA

**Keywords:** HIV, Nicotine, Brain, Spatial working memory, Contextual memory, Synaptic plasticity

## Abstract

**Background:**

Human immunodeficiency virus (HIV)-1-associated neurocognitive disorders (HAND) are characterized by synaptic damage and neuronal loss in the brain. Excessive glutamatergic transmission and loss of cholinergic neurons are the major indicators of HAND. Nicotine acts as a cholinergic channel modulator, and its cognitive-enhancing effect in neurodegenerative and cognitive disorders has been documented. However, it is unclear whether nicotine has any positive effect on memory and synaptic plasticity formation in HAND.

**Methods:**

We investigated the effects of nicotine on synaptic plasticity and hippocampus–prefrontal cortex (PFC)–amygdala-dependent memory formation in the HIV-1 transgenic (Tg) and F344 control rats.

**Results:**

Chronic nicotine treatment (0.4 mg/kg nicotine, base, subcutaneously) significantly attenuated the cognitive deficits in the HIV-1Tg rats in both the spatial and contextual fear memories but impaired the contextual learning memory in the F344 rats. To determine the role of nicotine in the synaptic dysfunction caused by HIV-1 proteins, we analyzed the expression of key representative genes related to synaptic plasticity in the hippocampus, PFC, and amygdala of the HIV-1Tg and F344 rats using a custom-designed qRT-PCR array. The HIV-1 proteins significantly altered the glutamate receptor-mediated intracellular calcium cascade and its downstream signaling cascade in a brain region-specific manner. Further, chronic nicotine treatment reversed the effect of HIV-1 proteins on the expression of genes involved in synaptic plasticity in the three brain regions. The effects of nicotine differed significantly in the HIV-1Tg and F344 rats.

**Conclusions:**

Our findings indicate that nicotine can attenuate the effect of HIV viral proteins on cognitive function and produce a brain region- and strain-specific effect on the intracellular signaling cascades involved in synaptic plasticity and memory formation.

**Electronic supplementary material:**

The online version of this article (doi:10.1186/s13041-015-0134-x) contains supplementary material, which is available to authorized users.

## Introduction

Human immunodeficiency virus (HIV)-1 proteins penetrate the central nervous system (CNS) during the early stages of viral infection and induce progressive neuronal damages in the brain [1, 2]. HIV-1-associated neurocognitive disorders (HAND) are the major CNS complications of HIV-1 infection, with approximately 50 % of patients developing some degrees of cognitive impairment [3]. Working (short-term) and episodic memory (involving short-term and long-term memory) impairments are the most commonly observed cognitive deficits in the HAND patients [3–5]. Recent studies have revealed that progression of HAND is accelerated by drug abuse in the HIV-1-infected patients. Cigarette smoking has a high prevalence in the HIV population, which abuses multiple drugs [6, 7]. It has been reasoned that the high prevalence of smoking in HIV-1-infected patients is attributable to the compensatory effect of smoking on cognitive deficits [7].

Nicotine is the main psychoactive component of cigarette smoke, and it has variable effects on cognitive function. There has been relatively little research on the relation between nicotine and progression of HAND. Recently, Wojna et al. [8] reported that HIV-1-infected smokers exhibited less cognitive impairment than infected non-smokers. Studies of animal models of HIV-1 infection demonstrate that chronic nicotine treatment restored the effect induced by viral proteins on the multiple intracellular signaling pathways in the different brain regions of the HIV-1Tg rats [9]. However, nicotine-induced molecular alterations in the neural circuits of working and episodic memory have not been fully elucidated in the presence of HIV-1 viral proteins.

Memory formation depends on the activity of interconnected neurons through synapses within the hippocampus, prefrontal cortex (PFC), and amygdala [10]. The dynamic interaction between glutamatergic and cholinergic synapses has a major role in encoding of new information into short-term and long-term memories [11, 12]. Dysfunction in the brain glutamatergic and cholinergic neurotransmission is postulated to be one of the contributors to memory impairment during HIV-1 infection [13, 14]. The effects of HIV-1 proteins on the glutamatergic neurotransmission system have been investigated [15, 16]. Results from these studies show that HIV viral proteins induce excessive glutamate release from infected microglia cells and result in glutamate-mediated neurotoxicity in the cortical and subcortical regions of the brain [16]. Further, mechanistic studies of HIV-1 infection have demonstrated that viral proteins lead to glutamate-induced deregulation of calcium homeostasis by overactivating N-Methyl-D-aspartic acid (NMDA) and metabotropic glutamate receptors (mGluRs). Activation of these receptors enhances subsequent calcium-mediated apoptosis signaling in the neurons [13, 17, 18]. Recent studies also showed that brain cholinergic neurons are affected by HIV viral proteins [13, 14]. Depboylu et al. [19] reported that chronic inflammation acted as a key mediator of HIV-1 protein-induced cholinergic dysfunction and neuronal loss in the brain that contributes to learning and memory impairment.

Stimulation of the cholinergic system by nicotinic acetylcholine receptor (nAChR) agonists protects against glutamate-mediated neurotoxicity and leads to subsequent cognitive improvement in rodent models of neurodegenerative disorders [20]. A low dose of nicotine enhances cognitive performance by attenuating the impairment of cholinergic neurotransmission [21, 22]. Experimental treatment of neurodegenerative disorders shows that nicotine mediates neuroprotection against NMDA-mediated excitotoxicity in the neurons by calcium-dependent mechanisms via neuronal α4β2- and α7-containing nAChRs [23–26]. These results indicate that nicotine has a modulatory effect on the neuronal circuitry of learning and memory during glutamate-mediated excitotoxicity and neurodegeneration.

The main goal of the current study was to determine how nicotine induces behavioral and molecular alterations in cognitive-impaired HIV-1Tg rats. From these experiments, we aimed to answer the following two interrelated questions: (1) does chronic nicotine treatment attenuate HIV-1-associated cognitive impairment? And (2) does nicotine show differential effects on intracellular signaling mechanisms underlying the synaptic plasticity formation of the HIV-1Tg rats in comparison to the F344 control animals? We addressed the first question by testing HIV-1Tg and F344 rats for spatial working memory in the Y-maze and then for contextual fear memory in the passive avoidance test. We then answered the question further by analyzing the transcription of genes involved in synaptic plasticity in the hippocampus, PFC, and amygdala of the same set of rats.

## Materials and methods

### Animals

Male HIV-1Tg and F344 genetic background control rats (Harlan Industries, USA) at 7 to 8 weeks of age were used. Rats were housed two per cage in a temperature (20 °C–22 °C)- and humidity (45 %–55 %)-controlled environment on a 12-h light/dark cycle. Food and water were provided *ad libitum*. All behavioral experiments were conducted between 9:00 am and 1:00 pm and were in accordance with the guideline of the University of Virginia Animal Research Committee.

### Drugs and treatment

Rats from each strain were divided randomly into two groups: saline-treated control and nicotine treated, designated as follows: F344_Saline (n = 11); F344_Nicotine (n = 12); HIV-1Tg_Saline (n = 9); HIV-1Tg_Nicotine (n = 11). To determine the effects of chronic nicotine treatment on spatial working and contextual memory, F344 and HIV-1Tg rats received a single subcutaneous injection of either saline or nicotine at a dose of 0.4 mg/kg/day (nicotine free base) for 15 days before starting the behavioral experiments.

(−)–Nicotine hydrogen tartrate (Sigma, St. Louis, MO) was dissolved in 0.9 % physiological saline, and its concentration was calculated as nicotine free base. On day 15, rats were tested for their spatial working memory in the Y-maze with the spontaneous alternation behavioral paradigm. Contextual fear memory experiments were conducted 1 week after the spontaneous alternation behavior test. Between days 22 and 26, rats were tested for short- and long-term contextual fear memory performance in the passive avoidance box with the one-step-through passive avoidance protocol. All rats received their last nicotine or saline injection on day 27 and were decapitated within 24 h after the last injection. Because of congenital cataracts of the HIV-1Tg rats, all behavioral experiments were conducted under dimmed red light to minimize the visual differences between the strains.

### Y–maze task test

Spatial working memory performance of HIV-1Tg and F344 rats was assessed by recording spontaneous alternation behavior during an 8-min session in the Y-maze test apparatus according to published protocols [27, 28]. The Y-maze has three arms, each 45 cm long, 10 cm wide, and 35 cm high. The arms were positioned at equal angles and converged in an equilateral triangular central area. Each rat was placed at the end of an arm and allowed to move freely through the Y-maze without reinforcements such as food, water, or electric shock. The series of arm entries was recorded by the Any-Maze video tracking system (Stoelting Co., Wood Dale, IL, USA). An arm entry was said to be complete when all four paws of the subject had been placed in the arm. Alternation behavior (actual alternations) was defined as successive entries into the three arms with overlapping triplet sets. The percent alternation was calculated as the ratio of actual alternation to possible alternations (total number of arm entries – 2) × 100.

### Passive avoidance test

The multiple trial one-step-through passive avoidance experiments were carried out between Days 22 and 26 with the goals of testing acquisition and short- and long-term contextual memory. The passive avoidance apparatus (Coulbourn Instruments Inc. Whitehall, PA, USA) consists of two compartments (illuminated and darkened) both equipped with a grid floor. The two compartments are separated by an automatic guillotine door.

The one-step-through passive avoidance experiment consisted of a single training and multiple testing sessions. On Day 24, each rat was placed in the illuminated chamber. After the placement, each rat explored the two compartments freely for 5 min. During the training session on Day 25, the rat was placed in the illuminated compartment for 15 s before the door was raised. After the rat entered the darkened compartment, the door was closed, and an electrical foot shock was delivered at 0.4 mA for 2 s. The subject was removed 30 s after receiving the foot shock and returned to its home cage. During the training session, retention latency for entering the dark compartment was recorded.

The test trials were conducted at the intervals of 1, 24, 48, and 72 h after the training session. During the test sessions, the rat was placed in the illuminated compartment, with the guillotine door closed for 30 s. After that, the door was opened, and retention latency for entering the dark compartment and the time spent in both compartments were recorded for 300 s, at which the time, the test was terminated. No electric shock was delivered during the test trials.

### Tissue collection

The animals were sacrificed and decapitated 1 day after the last nicotine injection, and their brains were immediately isolated. Tissues were collected from the hippocampus, PFC, and amygdala according to the rat brain atlas [29]. By using a rat brain matrix, slices of approximately 1.0 mm were taken from each brain, and tissues from the specific regions of interest were collected bilaterally from each slice with a 3.00-mm Harris Micro-Punch (GE Healthcare Life Sciences, Piscataway, NJ, USA). All punched tissue samples were stored at −80 °C until use.

### Quantitative RT-PCR (qRT-PCR) array

We used a custom-designed pathway-focused RT-PCR array with the goal of determining the RNA expression of genes involved in synaptic plasticity. By using the Ingenuity Pathway Analysis (IPA) software (http://ingenuity.com) to search the Kyoto Encyclopedia of Genes and Genome Pathway database (www.genome.jp/kegg/), we selected 80 genes related to synaptic plasticity formation for this custom-designed array. These genes can be grouped into the following biological processes: long-term potentiation (46 genes), long-term depression (25 genes), immediate early gene response (16 genes), cell adhesion (9 genes), extracellular matrix and proteolytic processing (2 genes), Creb signaling (20 genes), and postsynaptic density formation (16 genes).

The primers of each gene selected for the qRT-PCR assay were designed with Primer Express (v. 3.0) software (Applied Biosystems, Carlsbad, CA, USA) and spanned at least one intron to avoid amplifying genomic DNA. The primers had a melting temperature from 59 °C to 61 °C. Each pair of primers and their amplicon sequences were tested using the Basic Local Alignment Search Tool (BLAST; http://blast.ncbi.nlm.nih.gov/Blast.cgi) to ensure the specificity of the designed primers for the targeted genes. Dissociation curves were generated to check the specificity of the primers before including them in the qRT-PCR array. All primer sequences are listed in Additional file 1: Table S1.

Total RNA was isolated using the Trizol agent (Invitrogen, Carlsbad, CA, USA) according to the manufacturer’s protocol. The purity and quantity of total RNA were measured at optical densities of 260 and 280 nm with NanoDrop 2000c (Thermo Scientific, Waltham, MA, USA). Two μg of total RNA was reverse-transcribed into the first-strand cDNA using Superscript II reverse transcriptase. The cDNA mixture was incubated at 25 °C for 10 min, 42 °C for 1.5 h, and 70 °C for 15 min. The PCR amplification was conducted as described previously [30]. Briefly, the product was amplified in a volume of 10 μl containing 5.0 μl of 2× Power SYBR Green PCR Master Mix (Applied Biosystems) and combined sense and antisense primers (2.5 μl; final concentration 20 nM) in a 384-well plate using the 7900HT Sequence Detection System (Applied Biosystems). The PCR conditions were as follows: 95 °C for 10 min, followed by 40 cycles of 95 °C for 15 s and 60 °C for 1 min. A cycle threshold was assigned at the beginning of the logarithmic phase of the amplification, and differences in the *C*_*t*_ values of the control and nicotine-treated groups were used to determine the relative expression of genes of interest. Melting curve analysis was applied to characterize the specificity of the amplifications.

### Statistical analysis

Data are expressed as mean ± S.E.M. For the Y-maze experiments, data were analyzed using one-way analysis of variance (ANOVA) followed by post-hoc Bonferroni analysis. For the results of the passive avoidance test, data were analyzed using the Kruskall-Wallis ANOVA by comparing mean ranks followed by nonparametric analysis with the Mann–Whitney *U* test. The Kruskall Kruskall-Wallis test is a nonparametric H test; therefore, the significant result function definitions with this test are presented as H instead of parametric test script F. Differences were considered statistically significant at *p* < 0.05.

Expression of each gene of interest was first normalized to the expression of the housekeeping gene glyceraldehyde-3-phosphate dehydrogenase (GAPDH) and then analyzed using a comparative *C*_*t*_ method [31]. The relative expression of each gene was compared within and between HIV-1 Tg and F344 rats using the Student *t*-test. Significant alteration in mRNA expression was defined as a fold change > 20 % with a p value < 0.05 (N = 4–6 per group).

## Results

### Improvement of spatial working memory performance by chronic nicotine treatment in the HIV-1Tg and F344 rats

**Fig. 1:**
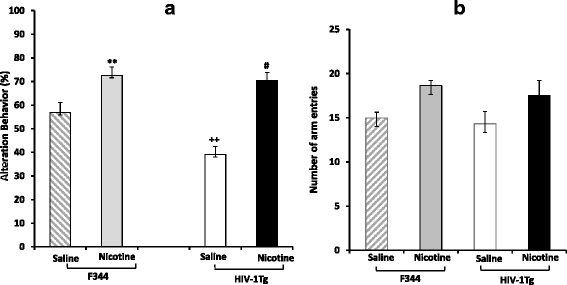
Nicotine effects on spatial working memory. **a** Spontaneous alteration behavior. **b** Number of arm entries during an eight-min session in the Y-maze task on day 15 (N = 9–12/group). Vertical bars show mean ± S.E.M. ^**, ++^ *p* < 0.01 compared with F344 saline-treated rats; ^#^ *p* < 0.01 compared with HIV-1Tg nicotine-treated rats

To evaluate the effects of HIV viral proteins and nicotine on spatial working memory performance, spontaneous alternation behavior was tested in the nicotine-treated and saline control groups of both HIV-1Tg and F344 rats. First, there was a significant group difference between the HIV-1Tg_Saline and F344_Saline groups (F [3, 31] = 10.83; *p* < 0.05) (Fig. 1a). Post hoc analysis revealed that the HIV-1Tg_Saline group showed a low frequency of spontaneous alternation behavior compared with the F344_Saline group (*p* < 0.01).

Chronic daily treatment with nicotine at a dose of 0.4 mg/kg for 15 days attenuated the viral protein-induced spatial working memory deficit. Spontaneous alternation behavior was significantly higher in the HIV-1Tg_Nicotine group than in the HIV-1Tg_Saline group (*p* < 0.01). No significant difference in the total number of arm entries was detected between the nicotine and control groups of either HIV-1Tg or F344 rats (Fig. 1b). These findings indicate that changes in the alternation behavior in the HIV-1Tg or F344 rats did not occur as a result of alterations in nicotine-induced locomotor activity.

### Chronic nicotine treatment enhanced the contextual memory in HIV-1Tg rats but impaired performance in F344 rats

**Fig. 2:**
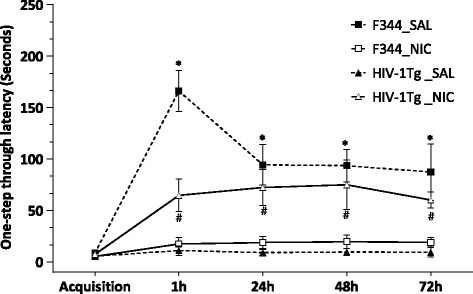
Effects of nicotine on step-through latency in the multiple-trial passive avoidance task on Days 22–26 in F344 and HIV-1Tg rats. Rats received daily nicotine injections five-min before the behavioral test (N = 9–12/group). Data are shown as mean ± S.E.M of the one-step through avoidance latencies at 1, 24, 48, and 72 h after the foot shock. *p < 0.05 in difference from F344-nicotine treated group; ^#^ *p* < 0.05 in difference from HIV-1Tg saline-treated group (Mann–Whitney *U*-test)

The effect of HIV-1 proteins and chronic nicotine treatment on the contextual memory performance was assessed by the one-step-through passive avoidance test in both rat strains. In the acquisition trial, we found no significant difference between the nicotine- and saline-treated groups of either F344 or HIV-1Tg rats (Fig. 2). However, there was a significant strain difference in the one-step-through latency in the 1, 24, 48, and 72 h retention trials (H = 9.12, *p* < 0.05; H = 14.74, *p* < 0.01; H = 7.89, *p* < 0.05; H = 8.50, *p* < 0.05, respectively). Nicotine prolonged the latency of entering the dark box in the HIV-1Tg rats, but impaired the passive avoidance behavior in F344 rats. Non-parametric analysis by the Mann–Whitney *U* test revealed that the HIV-1Tg_Nicotine group showed a significant increase in one-step-through latency at 24, 48, and 72 h compared with their control group (*p* < 0.05). In the F344 rats, chronic nicotine treatment induced a significant reduction in the one-step-through latency in the retention trials compared with the control group (*p* < 0.05).

### HIV-1 proteins altered the expression of synaptic plasticity genes in a brain region-specific manner

To determine the effect of HIV-1 proteins on the expression of genes related to synaptic plasticity, we analyzed the expression of the 80 genes in the qRT-PCR array. We found significant expression differences between the two strains on 16 genes in the hippocampus, 17 genes in the PFC, and 12 genes in the amygdala, respectively, which were classified into six signaling pathways in the hippocampus and five in the PFC or amygdala region of the HIV-1Tg_Saline group (Table 1).

**Table 1:** HIV-1 viral proteins induced significant alterations in the expression of synaptic plasticity genes. A total of 80 synaptic plasticity genes were analyzed using a qRT-PCR-array analysis. Among them, 50 showed significant strain-specific expression (*p* < 0.05 and fold change >20 %) in the hippocampus, prefrontal cortex, or amygdala

Signaling Pathway	Gene Name	Molecular Function	F344_Saline (Mean ± S.E.M)	HIV-1Tg_Saline (Mean ± S.E.M)	Ratio (HIV-1Tg/F344) Saline	*P* Value
Hippocampus						
Metabotropic glutamate receptor mediated PLC/IP3-dependent calcium signaling	Grm5	Metabotropic glutamate receptor activity	4.4E-02 ± 3.4E-03	6.0E-02 ± 2.1E-03	1.36	0.004
	Plcb4	Phospholipase C activity	2.4E-02 ± 2.2E-04	3.9E-04 ± 3.8E-04	1.63	0.005
	Itpr3	Calcium ion transmembrane transporter activity	7.3E-04 ± 1.5E-04	1.3E-03 ± 1.5E-04	1.78	0.02
Ca^+2^/calmodulin-dependent protein kinase signaling	Cacna1i	Voltage-gated calcium channel activity	2.0E-03 ± 3.6E-04	3.0E-03 ± 2.1E-04	1.5	0.04
	Camk4	Calmodulin dependent protein kinase activity	3.3E-02 ± 1.1E-03	3.9E-02 ± 1.7E-03	1.2	0.02
	Calm4	Calcium ion binding	2.6E-03 ± 3.1E-04	3.7E-03 ± 2.5E-04	1.42	0.03
CREB signaling	Creb-2 (Atf4)	Transcription factor activity	8.0E-03 ± 2.8E-04	1.2E-02 ± 4.4E-04	1.57	0.01
Kinase signaling	Akt1 (PKB)	Protein kinase activity	2.7E-02 ± 2.4E-03	4.0E-02 ± 2.6E-03	1.48	0.006
	Mapk3	Mitogen activated kinase activity	9.6E-02 ± 8.0E-03	7.2E-02 ± 6.5E-03	0.74	0.03
	Adcy8	Adenylate cyclase activity	7.6E-03 ± 4.2E-04	6.1E-03 ± 6.0E-04	0.80	0.033
Immediate early response gene	Arc	Actin binding	1.2E-02 ± 2.0E-02	6.2E-03 ± 1.2E-03	0.49	0.03
	Bdnf	Growth factor activity	4.2E-04 ± 5.0E-05	2.3E-04 ± 7.1E-05	0.56	0.02
	Jun-b	Transcription factor activity	1.5E-02 ± 2.2E-03	9.9E-03 ± 7.5E-04	0.63	0.03
	Ngf	Growth factor activity	6.5E-03 ± 4.7E-04	4.9E-03 ± 4.8E-04	0.75	0.01
Postsynaptic organization of synapse	Gria1	Glutamate receptor activity	2.2E-01 ± 2.4E-02	1.5E-01 ± 1.7E-02	0.69	0.006
	Adam10	Metallopeptidase activity	2.6E-02 ± 1.6E-03	1.9E-01 ± 2.0E-03	0.74	0.04
Prefrontal Cortex						
Metabotropic glutamate receptor mediated PLC/IP3-dependent calcium signaling	Grm1	Metabotropic glutamate receptor activity	5.6E-02 ± 8.0E-03	3.3E-02 ± 5.5E-03	0.58	0.03
	Plcb4	Phospholipase C activity	2.0E-02 ± 1.2E-04	1.4E-02 ± 5.8E-04	0.70	0.002
	Itpr1	Calcium Ion transmembrane transporter activity	7.0E-03 ± 7.9E-04	5.0E-03 ± 4.9E-04	0.71	0.04
	Itrp2	Calcium Ion transmembrane transporter activity	1.5E-01 ± 1.2E-02	1.2E-01 ± 1.5E-03	0.81	0.03
Ca^+2^/calmodulin-dependent kinase signaling	Cacna1g	Voltage–gated calcium channel activity	2.6E-02 ± 2.4E-03	1.7E-02 ± 1.0E-03	0.68	0.01
	Camk2g	Calmodulin-dependent kinase activity	2.6E-02 ± 1.8E-03	2.0E-02 ± 3.5E-04	0.79	0.02
	Cdh2	Calcium ion binding activity	6.9E-02 ± 1.2E-02	3.2E-02 ± 2.5E-03	0.46	0.01
Kinase signaling	Map2k1	MAP kinase activity	1.2E-01 ± 1.0E-02	9.2E-02 ± 4.2E-03	0.73	0.02
	Mapk3	MAP kinase activity	6.6E-02 ± 3.9E-03	5.4E-02 ± 2.1E-03	0.81	0.03
	Raf1	MAP kinase kinase activity	2.0E-02 ± 7.0E-04	1.6E-02 ± 1.0E-03	0.82	0.03
	Prkacb	Protein kinase A activity	1.9E-02 ± 1.3E-03	1.5E-02 ± 7.5E-04	0.79	0.04
	Akt1	Akt kinase activity	4.2E-02 ± 2.6E-03	3.4E-02 ± 1.6E-03	0.80	0.03
	JNK1	C-Jun N-terminal protein kinase	1.3E-01 ± 3.4E-03	1.9E-01 ± 4.3E-03	1.46	0.02
Inflammation-mediated neurodegenera-tive signaling	Il-6	Cytokine activity	1.5E-04 ± 1.5E-05	7.4E-05 ± 9.8E-06	0.48	0.002
	Ccl2	Chemokine activity	2.0E-04 ± 2.4E-05	1.2E-04 ± 1.2E-05	0.61	0.02
	Gabra5	GABA-A receptor activity	4.3E-02 ± 2.1E-03	3.3E-02 ± 2.5E-03	0.77	0.02
Postsynaptic density formation	Gria1	Glutamate receptor activity	8.4E-02 ± 4.5E-03	6.6E-02 ± 1.0E-03	0.79	0.02
Amygdala						
Ionotropic glutamate receptor- mediated Ca^+2^ signaling	Grin1	Ionotropic glutamate receptor activity	1.4E-01 ± 7.1E-01	1.0E-01 ± 8.3E-03	0.76	0.01
Ca^+2^/calmodulin-dependent kinase signaling	Cacna1g	Voltage–gated calcium channel activity	2.7E-01 ± 2.4E-03	1.7E-02 ± 5.0E-04	0.63	0.006
	Calm2	Calcium ion binding	1.0 ± 7.5E-02	7.4E-01 ± 2.8E-02	0.71	0.01
Kinase signaling	Mapk1	MAP kinase activity	2.7E-01 ± 2.2E-02	1.7E-01 ± 1.0E-03	0.61	0.02
	Mapk3	MAP kinase activity	9.3E-02 ± 9.4E-03	6.2E-02 ± 2.9E-03	0.67	0.01
	Raf1	MAP kinase kinase kinase activity	2.5E-02 ± 1.5E-03	1.7E-02 ± 8.6E-04	0.69	0.02
Immediate early gene response	Ntrk2	Growth factor activity	4.3E-03 ± 5.7E-04	2.5E-03 ± 2.1E-04	0.57	0.01
	Junb	Transcription factor activity	1.5E-02 ± 2.2E-03	9.9E-03 ± 7.6E-04	0.63	0.03
Postsynaptic organization of synapse	Gria3	Ionotropic glutamate receptor activity (AMPA)	4.1E-02 ± 3.5E-3	2.6E-02 ± 2.7E-03	0.63	0.02
	Adam10	Metallopeptidase activity	2.6E-02 ± 1.6E-03	1.9E-02 ± 2.0E-03	0.74	0.04
	Gria1	Ionotropic glutamate receptor activity (AMPA)	1.3E-01 ± 3.5E-03	1.0E-01 ± 5.3E-03	0.83	0.02
	Dlg4	Scaffold protein binding	2.5E-01 ± 2.9E-01	1.2E-01 ± 6.4E-03	0.46	0.003

**Fig. 3:**
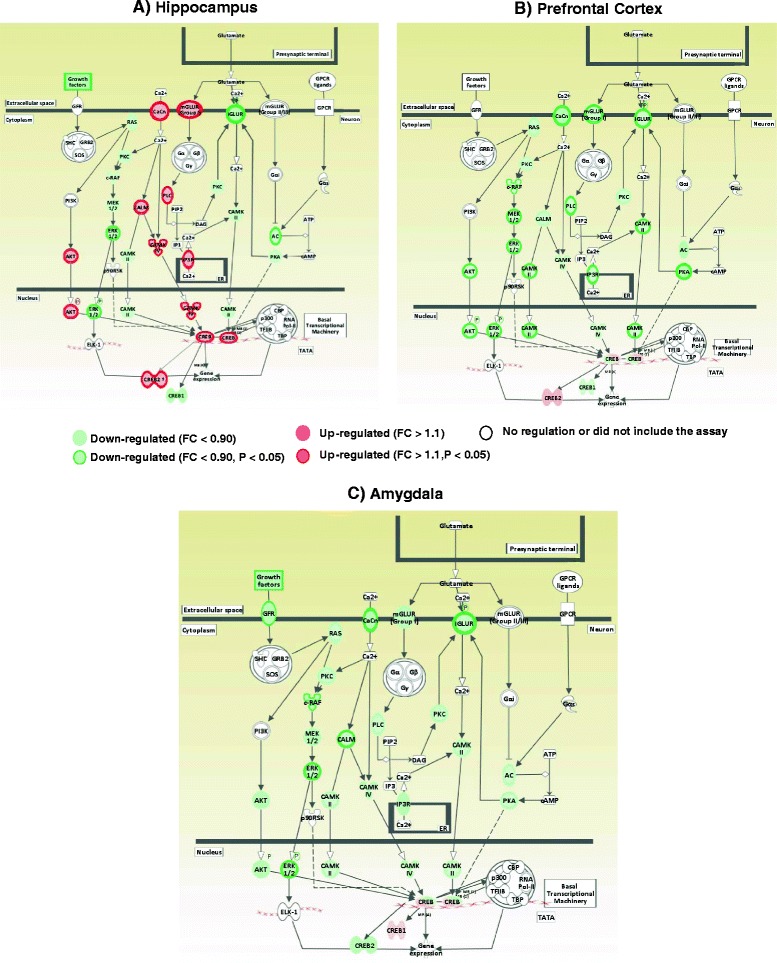
Effect of HIV-1 proteins on expression of genes in signaling cascades of synaptic plasticity formation in the hippocampus (**a**), prefrontal cortex (**b**), and amygdala (**c**). Genes involved in calcium signaling and its downstream targets were upregulated in the hippocampus but downregulated in the PFC and amygdala of HIV-1Tg rats compared with F344 control rats. The signaling cascades were drawn by IPA

The HIV-1 proteins altered the expression of genes encoding members of mGluR-mediated PLC/IP3-mediated calcium signaling, with 36 %–78 % significant upregulation of *Grm 5*, *Plcb4*, and *ltpr3* in the hippocampus and 19 %–30 % significant downregulation of *Grm1*, *Plcb4*, *Itpr1*, and *Itpr2* in the PFC (Table 1; Fig. 3a, b). In the voltage-dependent Ca^+ 2^ channel (VDCC)-mediated calcium signaling and Ca^+2^/calmodulin-dependent kinase signaling pathways, viral proteins significantly upregulated the expression of *Cacna1i*, *Calm4*, and *Camk4*, by 20 %–50 %, in the hippocampus but downregulated the expression of *Cacna1g* and *Camk2g* by 68 % and 79 %, respectively, in the PFC and of *Cacna1g* and *Calm2* by 63 % and 71 %, respectively, in the amygdala (Table 1). We did not observe significant alterations in response to viral proteins in the expression of genes involved in Creb signaling in the PFC and amygdala, but there was significant upregulation (43 %) of the expression of transcription factor Creb2 by viral proteins in the hippocampus (Fig. 3a).

Expression of genes involved in the MAP kinase signaling pathway and synapse formation was significantly changed by viral proteins in all three brain regions. In the MAP kinase pathway, viral proteins significantly decreased the expression of *Mapk3*, by 19 %, in the hippocampus; of *Raf1*, *Map2k1*, and *Mapk3*, by18 %–27 % in the PFC; and of the same genes, by 27 %–31 % in the amygdala. There was a significant decrease in the expression of genes associated with immediate early response and postsynaptic density formation in the hippocampus and amygdala (Table 1). Together, these results indicate that HIV-1 proteins exert a brain region-specific modulation of the expression of genes involved in intracellular calcium signaling and calmodulin-dependent kinase signaling pathways.

### Nicotine alters the synaptic plasticity gene expression in the cortico-limbic circuits of HIV-1Tg rats

Next, we wanted to determine how nicotine impacts the expression of genes involved in synaptic plasticity in HIV-1Tg rats. We found that nicotine significantly altered the expression of 22, 15, and 11 genes, respectively, in the hippocampus, PFC, and amygdala (Table 2). A gene ontology (GO) analysis of these genes showed that those significantly altered by nicotine can be classified into eight, five, and six signaling pathways in the hippocampus, PFC, and amygdala, respectively.

**Table 2:** Nicotine-induced alterations in expression of synaptic plasticity genes in the hippocampus, PFC, and amygdala of the HIV-1Tg rats. Genes exhibited significant alterations in the brain following chronic nicotine treatment in HIV-1Tg rats (*p* < 0.05 and fold ratio >20 %). Differential effects of nicotine on the gene expression determined by comparing the HIV-1Tg nicotine-treated group with HIV-1Tg saline group (Student’s *t*-test with Bonferroni correction)

Signaling Pathway	Gene Name	Molecular Function	HIV-1Tg_Saline (Mean ± S.E.M)	HIV-1Tg_Nicotine (Mean ± S.E.M)	(HIV-1Tg_Saline/ HIV-1Tg_Nicotine)	*P* Value
Hippocampus						
Ionotropic glutamate receptor- mediated Ca^+2^ influx	Grin2a	Ionotropic glutamate receptor	1.0E-01 ± 3.4E-03	9.1E-2 ± 4.9E-03	0.86	0.04
	Grin2b	Ionotropic glutamate receptor	2.8E-01 ± 5.1E-03	2.2E-1 ± 1.2E-02	0.88	0.04
Ca^+2^/calmodulin-dependent kinase signaling	Camk4	Calmodulin-dependent protein kinase activity	3.9E-02 ± 1.7E-03	3.2E-2 ± 1.9E-03	0.80	0.02
	Camk2g	Calmodulin-dependent protein kinase activity	2.5E-02 ± 5.8E-04	2.0E-02 ± 3.6E-04	0.80	0.03
PLC/IP3-mediated calcium signaling	Plcb4	Phospholipase C activity	3.9E-04 ± 3.8E-04	2.1E-04- ± 3.8E-03	0.52	0.007
	Itpr3	Calcium ion transmembrane transporter activity	1.3E-03 ± 1.5E-04	8.9E-04 ± 9.0E-05	0.64	0.03
	Itpr1	Calcium ion transmembrane transporter activity	7.2E-02 ± 4.2E-03	6.0E-02 ± 1.5E-03	0.84	0.03
CREB signaling	Creb-2 (Atf4)	Transcription factor activity	1.1E-02 ± 4.4E-04	8.3E-03 ± 4.2E-04	0.65	0.001
	Creb-1	Transcription factor activity	2.3E-02 ± 5.0E-04	3.4E-02 ± 1.8E-03	1.45	0.02
	Cbp	Transcription factor activity	3.3E-04 ± 2.5E-05	4.4E-04 ± 2.7E-05	1.34	0.01
Kinase signaling	Jnk1	C-Jun N-terminal protein kinase	1.2E-02 ± 5.2E-04	1.0E-02 ± 4.7E-04	0.85	0.04
	Mapk3	MAP kinase activity	7.2E-02 ± 6.5E-03	8.8E-02 ± 4.8E-03	1.22	0.03
	Adcy1	Adenylate cyclase activity	2.5E-01 ± 1.4E-02	3.2E-01 ± 7.0E-03	1.28	0.004
	Mapk1	MAP kinase activity	1.8E-01 ± 2.3E-02	2.4E-01 ± 4.2E-03	1.33	0.04
	Map2k1	MAP kinase activity	1.6E-01 ± 7.8E-03	2.2E-01 ± 7.3E-03	1.37	0.005
	Prkacb	Protein kinase A activity	9.5E-02 ± 2.0E-03	1.4E-01 ± 4.2E-03	1.53	0.001
	Kras	GTPase activity	3.3E-02 ± 2.8E-03	5.3E-02 ± 3.2E-03	1.62	0.002
Immediate early gene response	Nurr	Transcription factor activity	6.6E-03 ± 4.6E-04	8.2E-03 ± 5.6E-04	1.25	0.02
Postsynaptic organization of synapse	Synpo	Actin binding	4.9E-02 ± 2.5E-03	5.8E-02 ± 9.9E-04	1.14	0.04
	Gria2	Ionotropic glutamate receptor activity (AMPA)	1.6E-01 ± 2.1E-01	2.4E-01 ± 2.0E-02	1.55	0.03
Inflammation-mediated neurodegenerative signaling	Ccl2	Chemokine activity	1.2E-04 ± 1.3E-05	8.1E-5 ± 6.9E-06	0.63	0.02
Prefrontal Cortex						
Ionotropic glutamate receptor mediated Ca^+2^ influx	Grin2a	Ionotropic glutamate receptor	8.8E-02 ± 9.9E-03	5.1E-02 ± 2.9E-04	0.58	0.01
	Grin2c	Ionotropic glutamate receptor	1.0E-02 ± 8.2E-04	7.0E-03 ± 4.6E-04	0.64	0.001
Ca^+2^/calmodulin-dependent kinase signaling	Camk2b	Calmodulin-dependent protein kinase activity	0.1.7E-01 ± 1.0E-02	2.9E-01 ± 2.3E-03	1.67	0.004
	Camk4	Calmodulin-dependent protein kinase activity	3.1E-02 ± 2.0E-03	5.3E-02 ± 9.3E-04	1.69	0.001
	Cacna1i	Voltage–gated calcium channel activity	1.7E-02 ± 1.0E-03	3.2E-02 ± 1.7E-03	1.82	0.003
	Calm3	Calcium ion binding	6.0E-01 ± 1.1E-02	7.2E-01 ± 2.7E-02	1.14	0.01
PLC/IP3-mediated calcium signaling	Plcb3	Phospholipase C activity	2.2E-03 ± 2.4E-04	1.4E-03 ± 1.2E-04	0.65	0.01
	Itpr3	Calcium ion transmembrane transporter activity	4.5E-03 ± 2.5E-04	3.7E-03 ± 2.1E-04	0.82	0.02
Kinase signaling	Map2k2	MAP kinase activity	1.3E-03 ± 7.5E-05	9.3E-04 ± 1.4E-05	0.67	0.005
	Map2k1	MAP kinase activity	9.4E-02 ± 5.2E-03	6.5E-01 ± 3.2E-03	0.69	0.002
	Braf	MAP kinase kinase activity	3.1E-01 ± 1.0E-03	2.3E-02 ± 1.1E-03	0.75	0.001
	Mapk3	MAP kinase activity	5.4E-02 ± 2.1E-03	4.7E-02 ± 1.1E-03	0.86	0.04
	Akt1	Akt kinase activity	3.4E-02 ± 1.6E-03	5.2E-02 ± 5.2E-03	1.55	0.04
CREB signaling	Crem	Transcription factor activity	5.3E-03 ± 2.4E-04	3.8E-03 ± 4.1E-04	0.72	0.04
	Creb2	Transcription factor activity	1.5E-02 ± 8.5E-04	1.2E-02 ± 4.8E-04	0.83	0.03
Amygdala						
Ionotropic glutamate receptor mediated Ca^+2^ influx	Grin2d	Ionotropic glutamate receptor	1.9E-02 ± 1.9E-03	1.4E-02 ± 1.0E-03	0.76	0.04
	Grin2b	Ionotropic glutamate receptor	1.3E-01 ± 7.2E-03	1.0E-01 ± 7.3E-03	0.80	0.04
Ca^+2^/calmodulin-dependent kinase signaling	Cacna1g	Voltage–gated calcium channel activity	1.7E-02 ± 5.0E-04	2.7E-02 ± 2.4E-03	1.59	0.006
	Camk2a	Calmodulin-dependent protein kinase activity	1.4E-01 ± 3.6E-01	2.4E-01 ± 1.7E-01	1.66	0.001
	Camk4	Calmodulin-dependent protein kinase activity	1.5E-02 ± 1.0E-03	2.6E-03 ± 3.4E-03	1.72	0.02
PLC/IP3-mediated calcium signaling	Plcb4	Phospholipase C activity	2.3E-02 ± 2.3E-03	1.5E-02 ± 2.7E-03	0.64	0.04
	Calm2	Calcium ion binding	7.4E-01 ± 2.8E-02	5.9E-01 ± 1.9E-02	0.80	0.02
Kinase signaling	Kras	GTPase activity	4.1E-02 ± 2.8E-03	3.2E-03 ± 2.5E-03	0.77	0.04
	Prkacb	Protein kinase A activity	1.5E-01 ± 8.3E-03	2.4E-01 ± 1.3E-03	1.55	0.001
CREB signaling	Cbp	Transcription factor activity	3.3E-04 ± 2.5E-05	4.4E-04 ± 2.7E-05	1.34	0.01
Immediate early gene response	Mmp-9	Metallopeptidase activity	7.1E-05 ± 1.2E-05	1.3E-04 ± 2.0E-05	1.82	0.03

**Fig. 4:**
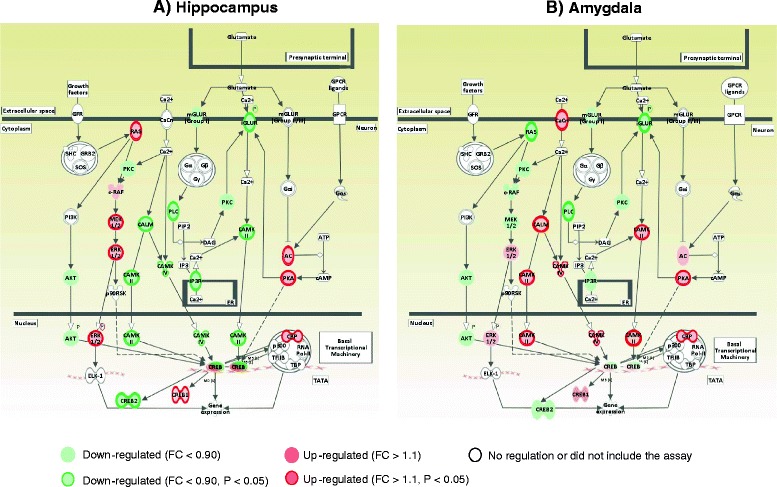
Synergistic effects of HIV-1 proteins and nicotine on the expression of genes in the signaling cascades of synaptic formation in the hippocampus (**a**) and amygdala (**a**). Chronic nicotine treatment attenuated the HIV-1 protein effect on the expression of genes involved in calcium signaling cascades. Genes encoding the upstream regulators and downstream targets of calcium signaling cascades were downregulated by nicotine in the hippocampus but were upregulated by nicotine in the amygdala compared with the HIV-1Tg saline-treated group. Signaling cascades were drawn by IPA

Chronic nicotine treatment significantly decreased the expression of *Plcb4*, *Itpr1*, and *Itpr3*, by 16 %–68 %, in the hippocampus, of *Plcb3* and *Itpr3*, by18 %–35 %, in the PFC, and of *Plcb4*, by 36 % in the amygdala (Fig. 4 and Additional file 2: Figure S1a). In the Ca^+2^/calmodulin-dependent kinase signaling pathway, nicotine significantly downregulated the expression of *Camk4* and *Camk2g*, by 20 %, in the hippocampus but upregulated the expression of *Camk2b*, *Camk4*, *Cancna1i*, and *Calm3*, by 14 %–82 %, in the PFC and of *Cacna1g*, *Camk2a*, and *Camk4*, by 59 %–72 %, in the amygdala. In Ras/MEK/ERK signaling pathway, nicotine significantly upregulated the expression of *Mapk3*, *Mapk1*, *Map2k1*, and *Kras*, by 22 %–67 %, in the hippocampus and downregulated the expression of *Map2k2*, *Map2k1*, *Mapk3*, and *Braf*, by 14 %–33 %, in the PFC and of *Kras*, by 23 %, in the amygdala (Table 2).

Consistent with the increased expression of hippocampal MAPK genes, expression of *Creb* and its cofactors was changed in nicotine-treated HIV-1Tg rats, with 34 %–45 % upregulation of *Creb1* and *Cebpb* in the hippocampus, 17 %–28 % downregulation of *Cebpb* and *Creb2* in the PFC, and 34 %–82 % upregulation of *Cebpb* and its target *MMP-9* in the amygdala (Table 2; Fig. 4a and b). Further, chronic nicotine treatment reversed the effect of viral proteins on the expression of *Creb2*, which was decreased by 35 % in the hippocampus of HIV-1Tg rats.

### Nicotine displays distinct effects on synaptic plasticity gene expression in F344 rats

The same set of genes was analyzed in the hippocampus, PFC, and amygdala of F344 rats in order to determine whether nicotine had the same effects on expression of the genes related to synaptic plasticity under normal physiological condition. Among the 80 genes examined, we found that 14 in the hippocampus, 18 in PFC, and 12 in the amygdala, respectively, were differentially expressed in the nicotine-treated and saline control groups (Table 3). The GO analysis showed that nicotine altered four, six, and four signaling pathways in the hippocampus, PFC, and amygdala, respectively (Table 3).

**Table 3:** Effect of nicotine on expression of genes involved in synaptic plasticity in the hippocampus, PFC, and amygdala of F344 rats. Significant expression alterations were determined by comparing the F344 nicotine-treated group with the F344 saline-treated group (Student’s *t*-test with Bonferroni correction). Significance set as *p* < 0.05 and fold ratio >20 %

Signaling Pathway	Gene Name	Molecular Function	F344_Saline (Mean ± S.E.M)	F344_Nicotine (Mean ± S.E.M)	Ratio (F344_Saline/F344_Nicotine)	*P* Value
Hippocampus						
Ca^+2^/calmodulin-dependent kinase signaling	Camk4	Calmodulin-dependent protein kinase activity	3.3E-02 ± 1.1E-03	5.2E-02 ± 9.4E-04	1.57	0.007
	Cacna1i	Voltage–gated calcium channel activity	2.0E-03 ± 3.6E-04	2.9E-03 ± 1.1E-04	1.46	0.03
PLC/IP3-mediated calcium signaling	Plcb4	Phospholipase C activity	2.4E-02 ± 2.2E-04	1.5E-02 ± 2.2E-03	0.66	0.02
Kinase signaling	Hras	GTPase activity	8.7E-06 ± 2.2E-06	4.1E-06 ± 1.7E-05	0.47	0.04
	Prkacb	Protein kinase A activity	1.9E-02 ± 1.3E-03	1.5E-02 ± 1.6E-03	0.82	0.02
	Adcy1	Adenylate cyclase activity	3.3E-01 ± 2.1E-02	2.6E-01 ± 2.1E-02	0.78	0.006
	Mapk1	MAP kinase activity	4.3E-01 ± 2.1E-02	5.4E-01 ± 4.3E-03	1.25	0.01
	Raf1	MAP kinase kinase activity	2.0E-02 ± 7.0E-04	3.0E-02 ± 2.1E-03	1.52	0.01
	Map2k2	MAP kinase activity	2.3E-03 ± 1.7E-04	4.0E-03 ± 2.4E-04	1.73	0.001
Immediate early response gene	Ntrk2	Neurotrophin receptor activity	2.0E-03 ± 3.0E-04	1.1E-03 ± 5.1E-05	0.53	0.04
	Egr1	Transcription factor activity	9.4E-02 ± 9.6E-03	5.3E-02 ± 5.7E-03	0.55	0.03
	Jun-b	Transcription factor activity	1.5E-02 ± 2.2E-03	9.0E-03 ± 1.3E-03	0.57	0.02
	Arc	Activity-regulated cytoskeleton-associated protein	1.2E-02 ± 2.0E-03	7.9E-03 ± 8.0E-04	0.63	0.03
	Mmp9	Metallopeptidase activity	9.6E-05 ± 1.3E-05	1.8E-04 ± 3.5E-05	1.87	0.02
Prefrontal Cortex						
Ionotropic glutamate receptor-mediated Ca^+2^ influx	Grin2c	Ionotropic glutamate receptor	1.1E-02 ± 1.1E-03	8.5E-03 ± 5.2E-04	0.72	0.04
	Grin2d	Ionotropic glutamate receptor	4.0E-03 ± 7.6E-05	3.0E-03 ± 1.0E-04	0.76	0.03
Ca^+2^/calmodulin-dependent kinase signaling	Camk2b	Calmodulin-dependent protein kinase activity	1.7E-01 ± 1.0E-02	2.9E-01 ± 2.3E-03	1.67	0.004
	Cacna1g	Voltage–gated calcium channel activity	2.6E-02 ± 2.4E-03	4.0E-02 ± 2.5E-03	1.56	0.004
	Camk4	Calmodulin-dependent protein kinase activity	3.3E-02 ± 2.1E-03	5.3E-02 ± 3.3E-03	1.60	0.002
	Calm2	Calcium ion binding	4.4E-01 ± 6.0E-02	7.8E-01 ± 3.9E-02	1.73	0.001
PLC/IP3-mediated calcium signaling	Itpr2	Calcium ion transmembrane transporter activity	7.0E-03 ± 7.9E-04	4.6E-03 ± 3.3E-04	0.66	0.04
	Plcb3	Phospholipase C activity	3.0E-03 ± 2.4E-04	2.0E-03 ± 2.6E-04	0.68	0.03
	Itpr1	Calcium ion transmembrane transporter activity	7.0E-03 ± 7.9E-04	5.1E-01 ± 6.9E-04	0.73	0.01
Kinase signaling	Raf1	MAP kinase kinase activity	2.0E-02 ± 7.0E-04	1.4E-02 ± 1.8E-03	0.72	0.03
	Mapk3	MAP kinase activity	6.6E-02 ± 3.9E-03	4.9E-02 ± 1.1E-03	0.74	0.006
	Prkacb	Protein kinase A activity	1.9E-02 ± 1.3E-03	2.3E-02 ± 4.7E-03	1.15	0.01
	Akt1	Akt kinase activity	4.2E-02 ± 2.6E-03	5.0E-02 ± 3.1E-03	1.26	0.02
	Adcy8	Adenylate cyclase activity	3.9E-03 ± 4.2E-04	5.1E-03 ± 2.4E-04	1.31	0.02
Immediate early response gene	Synpo	Actin binding	3.4E-02 ± 2.6E-03	4.4E-02 ± 2.8E-03	1.27	0.04
	Ngf	Growth factor activity	1.5E-03 ± 1.6E-04	1.9E-03 ± 7.2E-05	1.29	0.03
Inflammation-mediated neurodegenerative signaling	Il-6	Cytokine activity	1.5E-4 ± 1.5E-05	7.0E-05 ± 7.8E-06	0.45	0.001
	Tnf	Cytokine activity	4.2E-04 ± 1.1E-04	3.0E-04 ± 2.8E-05	0.70	0.04
Amygdala						
Ca^+2^/calmodulin-dependent kinase signaling	Calm1	Calcium ion binding	1.2 ± 9.0E-02	9.2E-01 ± 7.8E-02	0.75	0.04
	Camk2g	Calmodulin-dependent protein kinase activity	3.2E-2 ± 2.4E-03	2.5E-02 ± 1.6E-03	0.80	0.04
PLC/IP3-mediated calcium signaling	Itpr2	Calcium ion transmembrane transporter activity	6.8E-03 ± 4.2E-04	5.5E-03 ± 1.0E-04	0.80	0.02
	Itpr3	Calcium ion transmembrane transporter activity	3.6E-03 ± 3.4E-04	3.0E-03 ± 7.7E-05	0.82	0.03
Kinase signaling	Mapk1	MAP kinase activity	2.7E-01 ± 2.2E-02	1.5E-01 ± 2.0E-01	0.55	0.007
	Raf1	MAP kinase kinase activity	2.5E-02 ± 1.5E-03	1.8E-02 ± 2.0E-03	0.72	0.02
Immediate early response gene	Fos-b	Transcription factor activity	1.0E-02 ± 3.2E-03	3.8E-03 ± 9.7E-04	0.38	0.03
	Junb	Transcription factor activity	1.5E-02 ± 2.2E-0	8.0E-03 ± 1.4E-03	0.51	0.02
	Egr1	Transcription factor binding activity	9.4E-02 ± 9.7E-03	5.8E-02 ± 3.8E-03	0.61	0.01
	Ntrk2	Neurotrophin receptor activity	4.3E-03 ± 5.7E-04	2.6E-03 ± 1.9E-04	0.61	0.02
	Bdnf	Growth factor activity	9.2E-05 ± 5.3E-06	5.8E-05 ± 1.6E-05	0.63	0.03
	Fos	Transcription factor activity	3.0E-03 ± 5.0E-04	1.9E-03 ± 2.0E-04	0.63	0.04

**Fig. 5:**
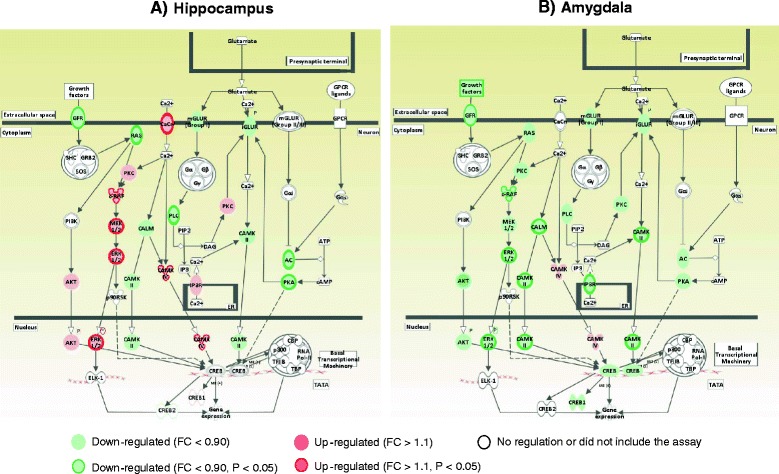
Regulation of synaptic plasticity gene expression by nicotine in the hippocampus (**a**) and amygdala (**b**) of F344 rats. Moderate chronic dose of nicotine increased expression of genes in the Ca^+2^/calmodulin-dependent kinase-IV signaling and Raf/Mek/Erk signaling pathways in the hippocampus. However, the same dose of nicotine downregulated the majority of synaptic plasticity genes in the amygdala compared with the F344 saline-treated group. Signaling cascades were drawn by IPA

The genes involved in PLC/IP3-mediated calcium signaling were significantly downregulated by nicotine in the three brain regions of F344 rats (*P* = 0.01–0.04) (Table 3). However, nicotine showed bidirectional effects on the expression of genes involved in VDCC-mediated calcium signaling and the Ca^+2^/calmodulin-dependent kinase signaling pathway, with 46 %–57 % upregulation of *Camk4* and *Cacnali* expression in the hippocampus, 20 %–25 % upregulation of *Calm1* and *Camk2g* expression in the amygdala, and 56 %–73 % downregulation of *Camk2b*, *Camk4*, *Calm2*, and *Cacna1g* expression in the PFC (Fig. 5 and Additional file 2: Figure S1b). The expression of genes involved in the Map kinase and AC/PKA signaling pathways was also significantly altered by nicotine in F344 rats. In the Raf/MEK/ERK signaling pathway, nicotine significantly downregulated the expression of *Raf1*, *Mapk1*, and *Map2k2*, by 25 %–73 %, in the hippocampus, of *Raf1* and *Mapk3*, by 72 % and 74 %, respectively, in the PFC, and of *Raf1*, by 72 %, and *Mapk1*, by 55 %, in the amygdala. In the AC/PKA signaling, nicotine significantly decreased the expression of *Adcy1*, by 22 %, and *Prkacb*, by 80 %, in the hippocampus but increased the expression of *Adcy1* by 26 % and of *Prkacb* by 15 % in the PFC (Table 3). We also observed nicotine-induced brain region-specific expression alterations in the immediate early gene response. For example, nicotine significantly upregulated expression of *Arc* and *Mmp-9* in the hippocampus and of *Ngf* and *Synpo* in the PFC but downregulated the expression of transcription factors Fos-b, Fos, Jun-b, and Egr1 and their downstream targets BDNF and Ntrk2 in the amygdala (Table 3 and Fig. 5b).

## Discussion

The current study provides detailed information on the effects of HIV-1 proteins and nicotine on expression of synaptic plasticity genes in the hippocampal–PFC–amygdala-dependent learning and memory mechanisms. The principal findings of this study are: (1) HIV-1 proteins impaired the hippocampal–PFC-dependent spatial working and hippocampal–amygdala-dependent contextual fear memory in HIV-1Tg rats (see Figs. 1 and 2); (2) the upstream regulators and downstream targets of intracellular calcium signaling were altered by HIV-1 proteins in a brain region-specific manner (see Fig. 3 and Table 1); (3) chronic nicotine treatment improved the spatial working and contextual fear memory performance in HIV-1Tg rats (Figs. 1 and 2) and attenuated the effect of viral proteins on synaptic plasticity formation in a brain region-specific manner (see Fig. 4 and Table 2); and (4) the effect of nicotine on the gene expression of synaptic plasticity and contextual fear memory formation differed significantly between the HIV-1Tg and F344 control rats. Nicotine impaired the contextual learning and related gene expression in the amygdala of the F344 rats (Fig. 2; Table 3). Together, these findings indicate that nicotine as a cholinergic channel modulator can attenuate HIV-1 effects on synaptic plasticity and memory formation, and the effect of hippocampal–amygdala-dependent contextual fear memory differed in the healthy (i.e., F344 rats) and disease (i.e., HIV-1Tg rats) states.

### HIV-1 proteins disturb synaptic plasticity formation in the hippocampus–PFC–amygdala circuit and impair the working and contextual memory in HIV-1Tg rats

Typically, HAND occurs in the early stages of HIV-1 infection and causes slow performance in the encoding and retrieval of spatial and contextual related information [5]. The HIV-1Tg rat expresses seven of the nine HIV genes and shows neurological and cognitive complications similar to those observed in HAND patients [32–34]. The non-infective HIV-1Tg rat offers a valid model for investigating the neuropathological aspects of memory impairment in HAND patients. Specifically, in this study, we showed that viral proteins induced a deficit in both spatial working and contextual memory performance in these animals. As shown in Figures 1a and 2, HIV-1Tg rats exhibited low spontaneous alteration behavior in the Y-maze and short latency in entering the dark box during the retention trials of the one-step-through passive avoidance test compared with the F344 control rats. Given that the HIV-1Tg rats show strong neuropathological similarities to HIV-1-infected patients with concomitant expression of multiple HIV genes, the observed memory deficits in HIV-1Tg rats are most likely the result of the neurodegenerative effects of multiple HIV-1 proteins within the neural circuits of learning and memory.

Glutamate-mediated excitotoxicity has been considered a central pathological mechanism for the effects of HIV-1 proteins on the neurodegenerative process in the brain [16]. These proteins lead to glutamate-induced deregulation of calcium homeostasis by over-activating the glutamate receptors and facilitating neurodegeneration in the brain [17, 18, 35, 36]. When an excessive amount of glutamate is released from the microglia and presynaptic neurons during the neurodegeneration, the calcium conductance of NMDA receptors is inhibited by intracellular calcium through calcium sensitive proteins [37]. The calcium-induced NMDA receptor inactivation serves as a negative feedback mechanism and results in downregulation of NMDA receptors [38–40]. Here, we demonstrated that HIV-1Tg rats showed significant downregulation of NMDA receptor subunits in the hippocampus, PFC and amygdala (Fig. 1). This result suggests the activation of the negative feedback loop, which could operate as a physiological homeostatic mechanism to limit the level of neurotoxicity caused by viral proteins. However, this type of protection may also limit the level of neuronal activity in HIV-1Tg rats that may contribute to spatial and contextual memory deficit. Further, our gene expression data showed the selective effects of viral proteins on the expression of genes in the intracellular calcium signaling pathways. As shown Figure 3a, viral proteins significantly upregulated the expression of genes involved in mGluR5-mediated calcium signaling but downregulated the expression of immediate early genes (IEGs) in the hippocampus. These results support previous studies, which showed that low expression of IEG mRNAs was associated with mGluR-dependent long-term depression (LTD) formation in the hippocampus [41–43]. However, HIV-1 proteins decreased calcium signaling in the PFC and amygdala by downregulating genes in the VDCC- and PLC/IP3-mediated calcium signaling pathways (Fig. 3b, c). Considerable evidence demonstrates that dysfunction in VDCC and IP3 receptor activity plays a central role in synaptic loss and memory impairment during neurodegeneration in the brain. In particular, reduction in calcium signaling inhibits onset of LTP and long-term memory acquisition through deactivation of calcium-sensitive Ras/ERK and CAMK signaling pathways [44, 45]. We confirmed the aforementioned findings in cognitively impaired HIV-1Tg rats by showing the low expression of VDCC and IP3 receptor genes with significant downregulation in the calcium-sensitive protein kinase signaling pathways in both the PFC and the amygdala (see Table 1).

The results presented here clearly show that HIV-1 proteins display a brain region-specific effect on calcium signaling, which may be considered an important factor causing synaptic plasticity dysfunction and memory impairment in HIV-1Tg rats. One possible explanation for this effect is that HIV-1 proteins enhance hippocampal LTD induction by overactivating mGluRs-PLC/IP3- mediated calcium signaling through excessive release of glutamate from the microglia cells. The viral proteins mediate this deregulation of calcium signaling and may facilitate the synaptic loss and reduce the hippocampal synaptic projections to the PFC and amygdala. Low hippocampal presynaptic stimulation of the PFC and amygdala circuits could reduce the LTP induction that is mediated by calcium entry through VDCC in the neurons of the PFC and amygdala. Further, in support of the link between hippocampal LTD and LTP formation in the PFC and amygdala of HIV-1Tg rats, blocking LTP induction in the hippocampus inhibits LTP formation in the medial PFC-basolateral amygdala [46]. These alterations are associated with memory impairment during brain neurodegeneration [47, 48].

### Chronic nicotine treatment attenuates the effects of HIV-1 proteins on synaptic plasticity and enhances working and contextual memory in HIV-1Tg rats

Nicotine as well as other nicotinic receptor agonists show neuroprotective effects against glutamate-mediated neurotoxicity and enhance cognitive performance in a variety of neurodegenerative disorders [25, 49, 50]. As such, this cognitive-enhancing effect of nicotine is defined as one of the main reasons that hinder efforts to quit smoking in HIV-1-infected patients [7]. Nicotine received through cigarette smoking in these patients preassumely increases the risk for HIV-1 infection related disease progression and mortality [7]. Therefore, determination of the molecular mechanisms underlying cognitive-enhancing and neuroprotective effects of nicotine in this particular population will provide new insights into potential drug treatment.

Nicotine can affect glutamatergic neurotransmission directly through nAChRs and indirectly through VDCC and IP3 receptors and modulate calcium signaling in glutamatergic neurons [51, 52]. However, the regulatory effects of nicotine on these calcium signaling pathways depend on the distribution of nAChRs and baseline intracellular calcium concentrations among the brain regions during neurodegeneration. Neuronal nicotinic acetylcholine receptors can contribute to modify the function of ionotropic glutamate receptors. Stimulation of α7 and β2 nicotinic acetylcholine receptors by cholinergic agonists decreases the surface expression of the NMDA receptor subunits during the glutamate toxicity. Shen et al. [53] reported that pre-treatment of nicotine abolished the glutamate-induced calcium influx by modulating surface expression of NMDA receptors through kinase signaling. Our results showed that chronic nicotine treatment significantly decreased expression of NMDA receptor subunits in the hippocampus, PFC, and amygdala of HIV-1Tg rats, as compared with their saline-treated counterparts (Fig. 4 and Additional file 2: Figure S1a). This suggests that nicotine may engage with negative feedback mechanisms in response to NMDA receptor activity-dependent calcium influx and contribute to limit the levels of neurotoxicity within neural circuits of learning and memory.

Our findings also showed that chronic nicotine treatment attenuated the effects of HIV-1 proteins on spatial working and contextual memory formation by selectively modulating the calcium-signaling pathways in the hippocampus, PFC, and amygdala. As shown in Figure 3 and Additional file 2: Figure S1a, nicotine significantly decreased the expression of IP3 receptor genes in the hippocampus and increased the expression of VDCC-mediated calcium signaling genes in the PFC and amygdala. Furthermore, we provide evidence for the nicotinic regulation of downstream protein kinase signaling pathways in the hippocampus, PFC, and amygdala of HIV-1Tg rats. Nicotine increased the expression of genes involved in MAPK and Creb signaling in the hippocampus and upregulated expression of CAMKs in the PFC and amygdala of HIV-1Tg rats (Table 2). These results indicate that nicotine shifted the formation of LTD into LTP induction in the hippocampus and promoted LTP expression in the PFC and amygdala, resulting in enhanced memory formation in HIV-1Tg rats. In contrast with our findings, Atluri and colleagues have demonstrated the negative effects of nicotine on the expression of synaptic plasticity gene expression and spine density in HIV-1 infected SK-N-MC cells [54]. Given the differences in the administration route of nicotine and different models of infection, an animal model of HIV-1 infection has more capability of assessing the mechanism of action of nicotine by providing detailed information in cognitively impaired HIV-1-infected patients. In fact, the observed positive effects of nicotine on the synaptic plasticity formation within the hippocampus, PFC, and amygdala circuits might be a secondary outcome caused by its anti-inflammatory effects in the central nervous system (CNS). Nicotine has been proved effective in suppressing inflammation in patients with Alzheimer and Parkinson diseases by modulating the cholinergic anti-inflammatory pathway, which contributes to cognitive enhancement in these patients. Recently, Depboylu et al. [19] reported that chronic inflammation induced by HIV-1 proteins reduced cholinergic transmission in the basal forebrain of rhesus macaques, suggested dysfunction of cholinergic anti-inflammatory pathway in the central nervous system. In this respect, the actions of nicotine in the anti-inflammatory system should be considered a factor in the findings from the present study that nicotine might also have an effect on microglia-dependent glutamate release, which could contribute to its modulatory effect in the neural circuits of learning and memory in HIV-1Tg rats.

### Nicotine shows differential effects on contextual memory formation in the health and disease states

In addition to the effects of nicotine on synaptic plasticity formation in HIV-1Tg rats, we analyzed the effects of nicotine in F344 rats to determine whether the drug exerts differential modulator effects on the synaptic plasticity and memory formation in health and disease states. When F344 rats were subjected to chronic nicotine treatment, the drug had different effects on spatial working memory and contextual fear memory. The drug enhanced the working memory performance in the Y-maze, whereas it impaired the contextual fear memory in the passive avoidance test (Figs. 1 and 2). This observation is consistent with previous reports [55, 56], where chronic nicotine treatment impaired short- and long-term formation of contextual fear memory. Here, we also showed that nicotine modulated gene expression in the hippocampus, PFC, and amygdala that was different from the effects in HIV-1Tg rats. Further, chronic nicotine treatment increased the expression of genes in the intracellular calcium signaling, Ca^+2^/calmodulin-dependent kinase, and IEG in the hippocampus and PFC, whereas it downregulated the majority of synaptic plasticity-related genes in the amygdala of F344 rats (Table 3). These results indicate that nicotine had positive effects on the hippocampus–PFC circuit-dependent spatial working memory but showed negative effects on the formation of long-term episodic memory by decreasing gene expression in the amygdala, which is a predominant neural component in the neural circuit of contextual fear memory formation.

## Conclusion

The prevalence of cigarette smoking among HIV-1 infected patients is high. It has been hypothesized that patients with HIV-1 infection use nicotine to compensate for HIV-1-related complications, including cognitive deficit. Our results indicate that HIV-1 proteins significantly disrupt synaptic plasticity formation by altering the expression of genes involved in intracellular calcium signaling pathways in a brain region-specific manner, which results in the deficit in spatial and contextual fear memory observed in HIV-1Tg rats. On the other hand, the effect of HIV-1 proteins is attenuated by chronic nicotine treatment in HIV-1Tg rats. Interestingly, the effect of nicotine on spatial and contextual memory performance appears to be different in the healthy and disease states. This may explain why nicotine shows different effects on the stimulation of neural circuits of learning and memory in these two states. Together, these results are helpful for elucidating the molecular mechanisms underlying the cognitive impairment in HIV-1-infected smokers.

## Additional files

Additional file 1: Table S1. List of primer sequences used in the study. Description and primer sequences of 80 candidate genes tested as potential synaptic plasticity formation genes in qRT-PCR array. Additional file 2: Regulation of synaptic plasticity gene expression by nicotine in the PFC of HIV-1Tg (A) and F344 (B) rats.
