# Supplementary material for: Nicotine attenuates the effect of HIV-1 proteins on the neural circuits of working and contextual memories
Source: Mol Brain. 2015 Jul 24;8:43. doi: 10.1186/s13041-015-0134-x (PMC4513611; doi:10.1186/s13041-015-0134-x)
Supplement: Additional file 1: Table S1. — List of primer sequences used in the study. Description and primer sequences of 80 candidate genes tested as potential synaptic plasticity formation genes in qRT-PCR array. [file 13041_2015_134_MOESM1_ESM.pdf]

Additional File 1 (Table S1): Description and primer sequences of 80 candidate genes tested as potential synaptic plasticity formation genes in qRT-PCR array**.** Abbreviations: LTP: long-term potentiation, LTD: long-term depression, IEG: immediate early response genes, ECM: extracellular matrix molecules

Gene Name	General Function	Synaptic Plasticity Function	Forward Primer Sequence (5'-3')	Reverse Primer Sequence (5'-3')
Adam10	Metallopeptidase activity	Cell adhesion, ECM, Postsynaptic density	TCCCAAGCCCAACTTTACAGA	TGCACATTGCCCATTAATGC
Adcy1	Adenylate cyclase activity	LTP	TGACAAGCGAAGGGCATTG	TGGTAGAGAATGACGAACGGTTT
Adcy8	Adenylate cyclase activity	LTP	GCCCTGGCTGACTTCTCTCTT	TGTTGAACGAATGCTTGTTGATC
Adora1	Adenosine receptor activity	Postsynaptic density	AAGATCCCTCTCCGGTACAA	TACCACAAGGGAGAGAATCCA
Akt1	Akt kinase activity	Creb signaling	CCTTACAGCCCTCAAGTACTCATTC	GGCGTACTCCATGACAAAGCA
Arc	Actin binding	IEG, Postsynaptic density	GGAGAACGACACCAGGTCTCA	GGTGGTACCCCTTCCAGACA
Bdnf	Growth factor activity	IEG, LTP	CAGGAGCGTGACAACAA	TCACCTGGTGGAACTCA
Braf	MAP kinase kinase activity	LTP, LTD	CAGGACGGAGAGAAGAAACCAA	TCCTCTCCAGTAAGCCAGGAAA
Cacna1g	Voltage–gated calcium channel activity	LTP, LTD	TCAGCTGCCTGTCAACTCTCA	GGCCAAGCGCTTCTTTCTG
Cacna1i	Voltage–gated calcium channel activity	LTP, LTD	CGGCGCCTGGAAAAGAA	GGTGCACATGGAGTGGATGA
Calm1	Calcium ion binding	Creb signaling, LTP	CGTGAGGCATTCCGAGTCTT	TGACGTGGCGCAGTTCTG
Calm2	Calcium ion binding	LTP	GCTGCAGGACATGATCAATGA	GGAATTCAGGGAAGTCGATTGT
Calm3	Calcium ion binding	LTP	AGGATGGGAATGGCTACATCAG	CCCCCAGGTTCGTCATGAC
Calm4	Calcium ion binding	LTP	AAGGAGCAGGTGGCTGAGTTAC	CGGCCATCCTTATTCTTGTCA
Camk2a	Calmodulin-dependent protein kinase activity	IEG, LTP, Postsynaptic density	GCATCTGCCGCTTGTTGAA	CTCGGAGATGCTGTCATGGA
Camk2b	Calmodulin-dependent protein kinase activity	IEG, LTP, Post synaptic density	ACCACAGAGCAGCTCATCGA	GGCCTGGGTCACAGATTTTC
Camk2g	Calmodulin-dependent	IEG, LTP	GCCAAGGTTGGTGAGCTCAA	TTGCCTGCACCCAGTTCTG

	protein kinase activity			
Camk4	Calmodulin-dependent protein kinase activity	IEG, LTP, Postsynaptic density	GAACCTCGTCCCGGATTACTG	TCGAAGAAATCGCTCAGAGGAT
Cbp	Transcription factor activity	Creb signaling	GGGTGGGATCTCAGCAATGT	CAGGTTTGTGTGCCGATTGT
Ccl2	Chemokine activity	LTP	CTCAGCCAGATGCAGTTAATGC	AGCCGACTCATTGGGATCAT
Cebpb	Transcription factor activity	Creb signaling	GAGCGCGCCATATTCAG	AGAGGTCGGAAAGGAAGT
Crem	Transcription factor activity	Creb signaling	CTGCTGCCACAGGTGACATG	CACCTTGTGGCAAAGCAGTAGT
Creb1	Transcription factor activity	Creb signaling, LTP, IEG	GTGGAGATGCTGCTGTAA	ATGGATACCTGGGCTAATG
Creb2	Transcription factor activity	Creb signaling, LTD, IEG	AGTTGTTATGGCGTCCTC	CTGCTTCCCTGTTCTTCA
Cxcr4	Chemokine activity	LTP	CCTTGCCCCAAGTCACATG	GGAGCTCGGATCTGGTAAGTTG
Dbh	Dopamine beta-monooxygenase activity, oxidoreductase activity	LTP	CAGGATCCTCCCGGTTTCTC	CGCCGGCCTTGTATATTCC
Dhcr24	Oxidoreductase activity	LTP	TCTGCCTGTGTTGCCTGAAC	ATGCCGGTGCCCATGAT
Dlg4	Scaffold protein binding	Postsynaptic density, Cell adhesion	CCCCAACATGGACTGTCTCTGT	GGGCGTGTCTTCATCTTGGT
Egfr	Growth factor activity	IEG	TCCGCTGTGGCTTGCATT	CGTTGCAAGAAGGCGTCTTC
Egr1	Transcription factor activity	IEG	CGAGCGAACAACCCTACGA	CGTTATTCAGAGCGATGTCAGAA
Fas	Transmembrane apoptosis signaling receptor activity	LTD	TGCACCTCGTGTGGACTTGA	CATTTGGTGTTGCTGGTTCGT
Fos	Transcription factor activity	Creb signaling, IEG	CATGGGCTCCCCTGTCAAC	TGGCACTAGAGACGGACAGATC
Fosb	Transcription factor activity	Creb signaling, IEG	CGTCGGAGGGAGCTGACA	GCTCTGCCTTTTCCTCTTCGA

Gabra5	GABA-A receptor activity	LTP	CATGCGCCTGACGATCTCT	TCCATCGGGAAGTCCTCAAG
Gapdh	Protein and microtubule binding	Housekeeping gene	AATGTATCCGTTGTGGATCTGACAT	CTCGGCCGCCTGCTT
Gria1	Glutamate AMPA receptor activity	LTP, LTD, Postsynaptic density	CAGGTGCGCTTCGAAGGTT	GTCCGGCGCCCTTTCT
Gria2	Glutamate AMPA receptor activity	LTP,LTD	ACCCCGGAAGATTGGGTACT	CCAGACGTGTCATTTCCTGATG
Gria3	Glutamate AMPA receptor activity	LTP, LTD, Postsynaptic density	CTTGTCTATGGAAGAGCTGATA	GAGATTCCCAGGCTCATAAA
Grin1	Glutamate NMDA receptor activity	LTP, Postsynaptic density	CACAGGAGCGGGTAAACAACA	TGAGTAGCTCGCCCATCATTC
Grin2a	Glutamate NMDA receptor activity	LTP, Cell adhesion, Creb signaling, Postsynaptic density	GCATCTGCCACAACGAGAAG	CCCGCCATGTTATCGATGTC
Grin2b	Glutamate NMDA receptor activity	LTP, Cell adhesion, Creb signaling, Postsynaptic density	CTGTCCGCCTAGAGGTTTGG	TGCGCTGGGCTTCATCTT
Grin2c	Glutamate NMDA receptor activity	LTP, Postsynaptic density	CTAGGAAGAACGGGCAGGAA	AGAGAACCTCCCCCTGCTATTC
Grin2d	Glutamate NMDA receptor activity	LTP	GTCTGCACAGGTACTTCATGAACAT	GGTTCACCAGAAAGCCATCCT
Grm1	Metabotropic glutamate receptor activity	LTD, LTP, Postsynaptic density	TTGGAAGTGATGGATGGG	TGACCTCTGGAGACTGAA
Grm5	Metabotropic glutamate receptor activity	LTD, LTP	CAAGACTTGCAACAGTTCTC	GGAGTCCATAAGCCATAGAATA
Hmox1	Heme oxygenase, immune response activity	LTP	GCTGACAGAGGAACACAA	TGCAGAGGTAGTATCTTGAAC
Hras	GTPase activity	LTP, LTD	GGTGGAGAACCCAAGAAG	CGTTAACCGCCATCTCC
Il-6	Cytokine activity	LTP	AGGGAGATCTTGGAAATGAGAAAA	TCATCGCTGTTCATACAATCAGAA

Itpr1	Calcium ion transmembrane transporter activity	LTD	GCAGAGGGATCTACGAATGGAT	GTACAACGCAACGGTCATCAA
Itpr2	Calcium ion transmembrane transporter activity	LTD	GGGCGACCAGCGCTATG	CGATGTACTCCTGGTTCTTCCTGT A
Itpr3	Calcium ion transmembrane transporter activity	LTD	AGAACGACCGCAGGTTTGTC	GTTGGGTTTGGTGACCATGAT
Jun	Transcription factor activity	Creb signaling, IEG	AATGGGCACATCACCACTACAC	TGCTCGTCGGTCACGTTCT
Junb	Transcription factor activity	Creb signaling, IEG	TGCGGACGGTTTTGTCAA	GCGTCACGTGGTTCATCTTG
Kras	GTPase activity	LTP, LTD	TACGATAGAGGACTCCTACAG	TTGCACTGTACTCCTCTTG
Map2k1	MAP kinase activity	LTD, Creb signaling	GCAGCTCATGGTACATGCTTTC	CCAGCCTGCGAAGTCTACCT
Map2k2	MAP kinase activity	LTD, Creb signaling	GCAGCTCATGGTACATGCTTTC	CCAGCCTGCGAAGTCTACCT
Mapk1	MAP kinase activity	LTP, LTD, Creb signaling	GCATGGTTTGTTCTGCTTAT	TCAGGGTTCTCTGACAGTA
Mapk3	MAP kinase activity	LTP, LTD, Creb signaling	GCCCTCCAATCTGCTT	GCAAGGCCAAAATCAC
Mecp2	Transcription factor activity	Creb signaling	GGGTCTCATGGAGGAA	GTTTGCCCTCTTTGTCT
Mmp9	Metallopeptidase activity	ECM, Cell adhesion	TCGAAGGCGACCTCAAGTG	TTCGGTGTAGCTTTGGATCCA
Ncam1	Neuron-neuron adhesion, neurite fasciculation and outgrowth	Cell adhesion	GACCAGAGAAGCAAGAGACTCTAG ATG	CAGGGAGGACACACGAGCAT
Ngf	Growth factor activity	IEG	ACTCTGAGGTGCATAGC	TGGGACATTGCTATCTGT
Ngfr	Growth factor activity	LTD	GGGCCTTGTGGCCTATATTG	GCGCCTTGTTTATTTTGTTTGC
Nras	GTPase activity	LTP, LTD	GGTCTGCGGAGTTTGAGATTTT	CTCCAACCACCACCAGTTTGT
Ntrk2	Neurotrophin receptor activity	LTP	TCCTGGTGGCCGTGAAGA	GCTTCGCGATGAAAGTCCTT

Nurr1	Transcription factor activity	IEG	TGCCTTCTCCTGCATTGCT	GTTCCTTGAGCCCGTGTCTCT
Pick1	Protein kinase C activity	Postsynaptic density	CAGCTGATGCCCTGGGTCTA	CCGCTTTACCAACCCATCAT
Plcb3	Phospholipase C activity	LTP, LTD	GCCTCCCGGAACACGTT	TGGTTCACCTGCAGCTTCAG
Plcb4	Phospholipase C activity	LTD	CCTGATGGATTTGGAGATATTG	GCCCATTGCTCTCATTTG
Prkaca	Protein kinase A activity	LTP	TGGGACCCCCGAGTACTTG	ACAGCTTTGTTGTAGCCTTTGCT
Prkacb	Protein kinase A activity	LTP, Creb signaling	GAACCCTCCCCCGAGTAATG	GTTCCAAGGGTTTTTTCCTTTC
Prkca	Protein kinase C activity	LTP, LTD	CAAGTTTGCTGTTTTGTGGTTCA	GCACCCGGACAAGAGAAAGTAA
Prkcg	Protein kinase C activity	LTP	AGCAACTGGGCAAGTTTAAGGA	GAGGCCTATGGCGATTTCC
Raf1	MAP kinase kinase activity	LTP, LTD	ACATTGCCCGGCAGACA	TCTGTGGATGATGTTCTTTGCAT
Rps6ka1	MAP kinase activity	Creb signaling	CCCAACATCATCACCCTGAAA	TCAGCTCTGTCACCAGGTATACG
Sst	Growth hormone inhibition	LTP	CCCAGACTCCGTCAGTTTCTG	CTTGGCCAGTTCCTGTTTCC
Syn1	Actin binding	Postsynaptic density, Cell adhesion	ACGATGTGCGTGTCCAGAAG	TCCCTGACACTGATGTCCTCAT
Synpo	Actin binding	Postsynaptic density, Cell adhesion	CGCCAGTCACGGATGGA	AAGGGCAGCGAGCCAATT
Th	Catecholamine biosynthesis	LTP	TGTTGGCTGACCGCACAT	CCCAGAGATGCAAGTCCAATG
Tnf	Cytokine activity	Cell adhesion	CCTCAGCCTCTTCTCATT	GGAACTTCTCCTCCTTGT
