# Supplementary material for: Nicotine attenuates the effect of HIV-1 proteins on the neural circuits of working and contextual memories
Source: Mol Brain. 2015 Jul 24;8:43. doi: 10.1186/s13041-015-0134-x (PMC4513611; doi:10.1186/s13041-015-0134-x)
Supplement: Additional file 2: — Regulation of synaptic plasticity gene expression by nicotine in the PFC of HIV-1Tg (A) and F344 (B) rats. [file 13041_2015_134_MOESM2_ESM.pptx]

## Slide 1
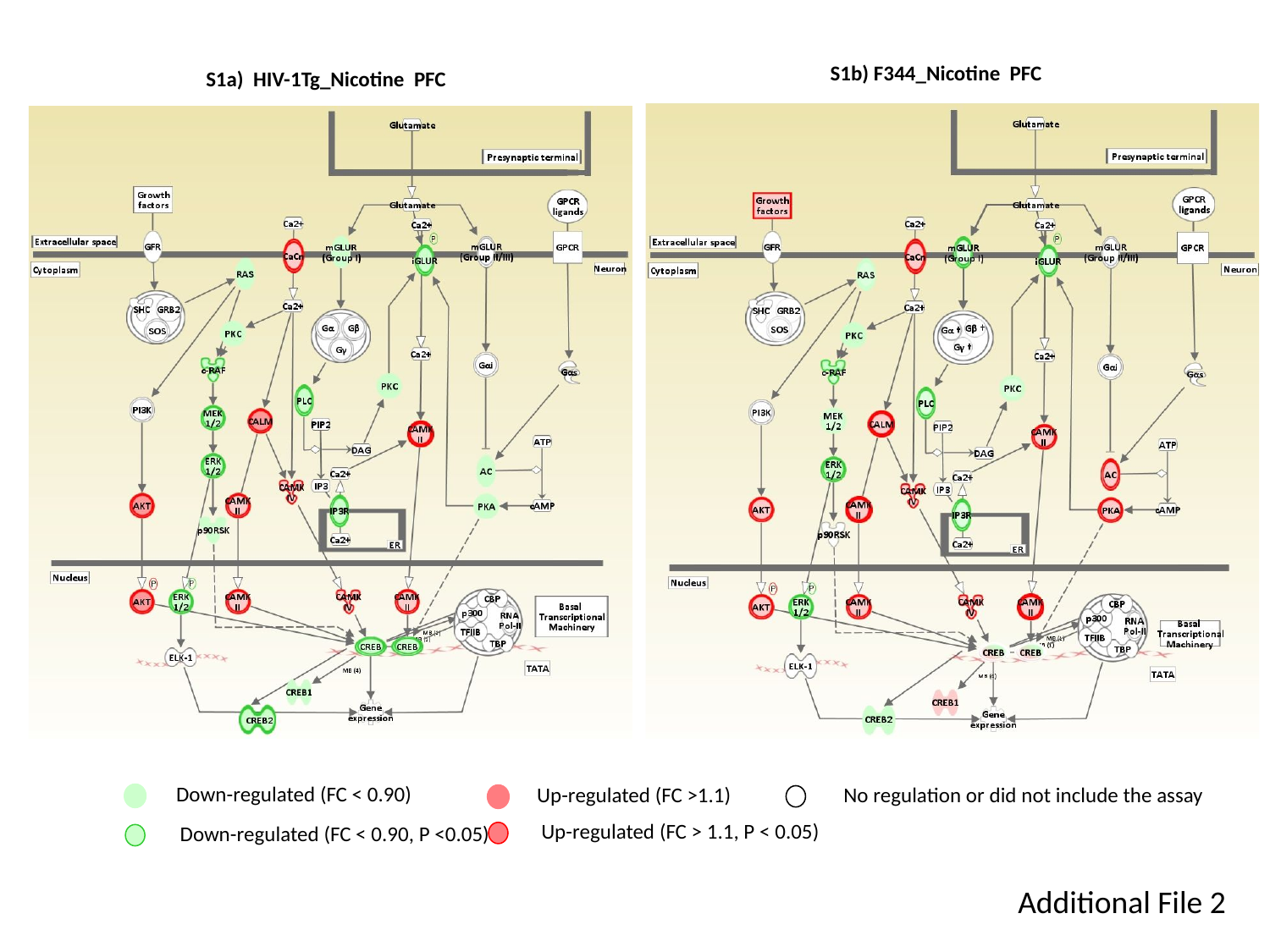

S1b) F344_Nicotine PFC
S1a) HIV-1Tg_Nicotine PFC
Down-regulated (FC < 0.90)
No regulation or did not include the assay
Up-regulated (FC >1.1)
Up-regulated (FC > 1.1, P < 0.05)
Down-regulated (FC < 0.90, P <0.05)
Additional File 2
